# Preparation and Characterization of a Novel Vinyl Polysiloxane Getter for Hydrogen Elimination

**DOI:** 10.3390/ma14081853

**Published:** 2021-04-08

**Authors:** Tao Xing, Yong Xu, Juying Wu, Yu Wang, Lifeng Yan

**Affiliations:** 1 Institute of System and Engineering, China Academy of Engineering Physics, 64 Mianshan Road, Mianyang 621900, China; xuy@caep.cn (Y.X.); wujy@caep.cn (J.W.); 2 Department of Chemical Physics, University of Science and Technology of China, Hefei 230026, China; wy1998@mai.ustc.edu.cn

**Keywords:** polysiloxane, hydrogen getter, uptake capacity, kinetics

## Abstract

Hydrogen generation and accumulation in confined spaces poses safety concerns due to its reactivity with oxygen to form explosions and the ability to embrittle metals. Various organic getters have been developed to eliminate hydrogen and minimize these undesired effects; however, these getters are usually powders with low molecular weights and are difficult to apply in complex structures. Polymer getters exhibit the promising features required for confined space applications, where could be readily processed into various shapes and forms. Unfortunately, polymer getters are relatively unexplored and their recorded performances are far from satisfactory. In this work, we report the preparation and characterization of novel vinyl polysiloxane getters. Starting from a methyl vinyl silicone oil prepared by ring-opening polymerization, polysiloxane getters in versatile forms that are adaptable to various environments are prepared by adding Pd/C and then curing. Combined with the thermal and radiation stability of polysiloxane, not only will these new getters be applicable in future applications in the electronic and nuclear industries as hydrogen scavengers, they also serve as platform for further development of polymer getters with superior properties.

## 1. Introduction

Hydrogen and its isotopes are highly flammable and are known to form explosive mixtures when their concentration in air reaches beyond 4% (volume fraction) [1]. The accumulation of hydrogen is detrimental in confined spaces where diffusion is limited. In various devices, such as batteries and electronics, internal concentrations of hydrogen needs to be below a few percent in order to be safe [2]. While both metal [3,4,5] and organic getters [2] have been developed and are known to be able to scavenge hydrogen, organic getters present better features in low concentration conditions because of their fast and irreversible nature of reaction. The most successful and widely used organic getters up to now have been mixtures of the alkyne (1,6-diphenoxy-2,4-hexadiyne (DPPE) and 1,4-bis(phenylethynyl)benzene (DEB)) and a noble metal catalyst [1,6]. The hydrogenation of these alkynes in the presence of precious metal is fast and irreversible [7,8], which makes them ideal for scenarios where hydrogen release occurs from slow and continuous sources (e.g., the radiolytic decomposition of moisture). Organic getters of this type are usually free-flowing powders with a density of about 0.6 g/mL. [9,10]. Powdered getters are difficult to handle and are usually bound into pellets to make them adaptable for different purposes; however, pellets are still brittle and incompatible with environments that feature complex shapes and sizes. Polymer getters would find better use in these conditions due to their processibility, where they can be readily manufactured into various shapes and forms [1,11,12]; however, polymer getters are relatively unexplored and their recorded performance is far from perfect. Sheppod et al. reported the preparation and characterization of a polymer getter based on polybutadiene. Both a getter pellet and elastomer were prepared and the pellets were used as hydrogen scavengers with transuranic waste [10,11]. By adding DEB into polydimethylsiloxane (PDMS), both bulk- and foam-type getters have been prepared [1,13,14]. With the 3D printing of a PDMS/DEB getter solution, Acosta et al. prepared a foam-type PDMS getter [1]. Printed getters feature superior uptake performance compared with cast getters due to their high surface area; however, due to the rheological constraints of 3D printing, only a minimal (4.3 wt %) DEB getter amount may be added to prepare the ink, and the resulting foam is not likely to feature high capacity. The commercially available PDMS getter from Honeywell [14] is a composite foam with a theoretical capacity lower than 100 g/mL. The cast getter reported by Balooch et al. [13] is composed of a 40 wt % DEB getter dispersed in a PDMS matrix and has a theoretical capacity of about 100 mL/g. Relatively lower capacities of the above getters make them unsuitable in specialized nuclear devices where limited space is available for getter installation. Another issue to be addressed when designing polymer getters is the stability duration, which is especially important in nuclear industry where radiation is usually present. PDMS has a unique Si-O-Si backbone and is resistant to aging and degradation caused by heat, radiation, and atomic oxygen [15]. Compared with polybutadiene, PDMS is a better choice when designing a polymer getter; however, the capacity of the PDMS getter reported so far is restricted to the ratio of the DEB getter, since the PDMS matrix is inert (i.e., not hydrogen reactive), which makes it difficult to obtain a mechanically flexible getter with a high uptake capacity.

In this study, we report the preparation and characterization of novel vinyl polysiloxane getters. Vinyl silicone oils were first prepared via anionic ring-opening polymerization of mixtures of octamethylcyclotetrasiloxane (D_4_^Me^) and 2,4,6,8-Tetravinyl-2,4,6,8-tetramethylcyclotetrasiloxane (D_4_^vi^), then blending them with a Pd/C catalyst to obtain pastes with high reactivity toward hydrogen. The pastes were further compounded with DEB and cured to provide getter elastomers with different compositions and capacities. Unlike the PDMS getters reported so far, which all use inert dimethyl silicone oil as a matrix, the methyl vinyl silicone oil employed in our work is reactive to hydrogen in the presence of a Pd/C catalyst and has a theoretical uptake capacity of 258 mL/g. With this additional hydrogen consumption from the polymer matrix, mechanical flexible polymer getters with a high uptake capacity could easily be prepared via compounding with a DEB getter. These new getters are characterized by flexibility, stability, and high capacity and are ideal hydrogen scavengers in various radioactive spaces with complex structures.

## 2. Methods

### 2.1. Materials

Tetramethylammonium hydroxide, octamethylcyclotetrasiloxane (D_4_^Me^), and 2,4,6,8-Tetravinyl-2,4,6,8-tetramethylcyclotetrasiloxane (D_4_^vi^) were sourced from J&K Chemical Inc (Beijing, China). IBr (Shanghai, China) and Na_2_S_2_O_3_ were sourced from Inorganic Chemical Inc (Beijing, China). All other chemicals were from sourced from Macklin Chemical Inc. (Shanghai, China) and were used as received without further purification.

### 2.2. Preparation and Characterization of Methyl Vinyl Silicone Oil

For the preparation of methyl viny silicone oil, 20 mg of N(Me)_4_OH was added to a 250 mL round bottom flame-dried flask. The catalyst was dried in a vacuum at 100 °C for 2 h. The vacuum was then broken by the addition of nitrogen, a mixture of 30 g D_4_^Me^, 30 g of D_4_^vi^, and 0.2 g of divinyl tetramethyl disiloxane added via a syringe. The mixture was heated to 110 °C and stirred overnight. The catalyst was destroyed by heating the mixture to 160 °C for 1 h at the end of the reaction. The reaction mixture was then cooled to room temperature and 150 mL toluene was added. The mixture was then poured into methanol. This process was repeated twice and the final clear oil obtained was dried in a high vacuum (1 mbar) at 100 °C for 8 h, where the product was numbered P2. Products P3 and P1 were prepared by the same procedure described above by adjusting the feed ratio of D_4_^vi^ to 100 wt % (P3) and 25 wt % (P1) while keeping the total weights of the two monomers at 60 g.

### 2.3. Vinyl Content Quantification

For vinyl content quantification [16], 40 mg of the vinyl silicone oil was dissolved in 5 mL of carbon tetrachloride in a 10-mL vial. Then, 0.4 mL of an iodine monobromide solution (2 mmol/mL in CCl_4_) was added to the silicone oil solution and the obtained solution was stirred at room temperature for 120 min. At the end of reaction, 4 mL of a KI aqueous solution (0.2 mmol/mL) was added and the obtained solution was shaken for 15 min. One drop of aqueous starch solution was added as an indicator and the mixture was titrated with a 0.2 mmol/mL Na_2_S_2_O_3_ solution. The Na_2_S_2_O_3_ solution consumed at the end of titration (when the color of the mixture turned from blue to colorless) was recorded. A blank test without the sample was also performed and the consumed Na_2_S_2_O_3_ solution was subtracted from sample consumption to give the actual consumption for each sample. Each sample was titrated three times and the average was used to calculate the vinyl content.

### 2.4. Getter Preparation

The DEB getter was prepared by dissolving 7.0 g of DEB into 100 mL of tetrahydrofuran at 65 °C. Then, 3.0 g of Pd/C (15 wt % Pd dispersed on porous carbon) was added and the mixture was evaporated to remove THF. The obtained black powder was dried in a vacuum at 55 °C for 4 h. The capacity of the getter was measured to be 216 mL/g according to the method described in Section 2.6.

The getter polymer (PH*x*, *x* = 1, 2, 3) was prepared by mixing silicone oils P1-P3 and Pd/C at a 3:1 weight ratio manually until a homogeneous mixture was obtained.

The cured getter (EH*x*, *x* = 1, 2, 3) was prepared via the extensive mixing of 0.8 g of silicone oil, 0.2 g of Pd/C, 20 mg of polyhydromethylsiloxane, and 8 μL of a Karstedt catalyst. The resulting thick paste was poured on a glass plate and cured at 65 °C for 3 days. The resulting soft elastomer was peeled off with a razor blade and further dried at 100 °C for 5 h.

The compounded getter (CG) was prepared by homogeneously mixing the DEB getter with P_3_ at ratios of 70 wt % (CG3), 60 wt % (CG2), and 50 wt % (CG1) and curing the mixtures with a 0.1 wt % Karstedt catalyst (2 wt % in xylene) at 55 °C overnight.

### 2.5. Characterization

Gel permeation chromatography (GPC) was performed with a Shimadzu GPC (Shimadzu Corporation, Kyoto, Japan) equipped with a DA-20 pump and a RID-10A refractive detector (Shimadzu Corporation, Kyoto, Japan). A sample of 3 mg/mL was prepared in THF and filtered through a 220-nm membrane before the test. All tests were performed at 35 °C and molecular weight and polydisperse index values were calculated with monodispersed PDMS from American Standard Corporation (Piscataway, NJ, USA). The ^1^H-NMR analysis was conducted on a 400 MHz Bruker (Billerica, MA, USA) spectrophotometer and the residual solvent peak (CDCl_3_ 7.26 ppm [^1^H]) was used as an internal chemical shift reference. Stress–strain tests were carried out with a Shimadzu EZ-SX (Shimadzu, Kyoto, Japan) tensile testing machine according to the standard procedures described in GB/T 528-92, where a type-II dog bone shape sample was used. Thermal stability of the cured getter was evaluated with a STA449F3 simultaneous thermal analyzer (NETZSCH, Waldkraiburg, Germany). Tests were conducted from room temperature to 600 °C in N_2_ or air at a heating rate of 10 °C/min. Gas chromatography tests were performed on a Shimadzu GC-2018 gas chromatograph equipped with a Shimadzu TDX-01 (Shimadzu, Kyoto, Japan) capillary column (1 m × 3 mm, pore size 1.5–2.0 nm) and TCD (Thermal Conductivity Detector) detector. To calculate the concentration of hydrogen in each sample, a calibration curve was first constructed using a standard sample with a known H_2_ concentration. The hydrogen peaks with a retention time of 1.2 min for each spectrum were integrated and plotted against concentration. Linear fitting of the results generated an equation that was then used to calculate the H_2_ concentration of sample gas.

### 2.6. Absorption Test in Pure H_2_

Absorption tests in a pure H_2_ atmosphere were performed on the homemade apparatus shown in Figure S1. The leakage rate of the apparatus was determined to be less than 10^–9^ Pa m^3^/s by an L300500i helium mass spectrometer leak detector (Leybold, Koln, Germany), which was more than appropriate for the test. The sample was placed in a stainless-steel reactor (Xuwei technology Inc., Chengdu, China) with a 100 mL volume, where the reactor was then closed and a vacuum was applied to the apparatus (valves 1 off, and 2, 3, and 4 on). After the internal pressure reached approximately 100 Pa, the vacuum pump and reactor were disconnected (valves 4 and 3 off) and H_2_ from the hydrogen tank was slowly admitted to the apparatus (valves 1 and 2 on) until the internal pressure reached 200 KPa. Data acquisition software (SUPY1.0, 2015, Simingte Inc., Hangzhou, China) was then used and the sample container was reconnected (valve 1 off and 3 on) and H_2_ was introduced into the reactor. Pressure change was recorded as a function of time and used to calculate capacity (volume of hydrogen absorbed per weight of getter, mL/g) based on the pressure drop according to the ideal gas equation (Equation (1)):

(1) $$\mathrm{Capacity}=\frac{\left(P_{1}V_{1}-P_{2}\left(V_{1}+V_{2}-V_{3}\right)\right)*22400}{mRT}$$

where *T* is temperature, m is the weight of the sample, R is a gas constant, *V*_1_ is the volume of the gas reservoir and tubing between valve 1 and two containers (gas reservoir and reactor), which was calibrated to be 312.0 mL. *V*_2_ is the volume of the reactor, which was calibrated to be 114.6 mL, *V*_3_ is the volume of the sample, which was about 0.8 mL when a 1.0 g sample was used.

### 2.7. Absorption Test in the Mixture Gas

Measurements were taken with the homemade apparatus shown in Figure S2. The sample was placed in a container with a volume of 100 mL and vacuumed to 20 Pa before the addition of the mixture gas with 1% or 5% H_2_ in N_2_ to a pressure of approximately 100 KPa. Sampling (20 μL) from the reactor was performed at a predetermined time interval and was analyzed with gas chromatography (GC-2018, Shimadzu Inc., Kyoto, Japan) to determine the hydrogen concentration.

### 2.8. Kinetic Study

Absorption tests at temperatures from 0 to 60 °C were performed in 1% H_2_ in N_2_ using a 0.3-g sample. For paste samples PH1-PH3, a 10-mL vial was used to load each sample. For elastomer getters EH1-EH3, thin films of about 300-μm in thickness were used. The H_2_ concentration was determined by GC and was considered with a first-order reaction equation (Equation (2)):

(2) $$kt=\ln\frac{1}{1-x}+C$$

where *t* is time, *k* is the rate constant, *x* is the ratio of consumed H_2,_ and C is the integration constant. For all samples tested, the linear correlation of *t* with $\ln\frac{1}{1-x}$ was observed when the H_2_ consumption is lower than about 30%, where deviation was observed with higher H_2_ consumption. Only the linear data were fitted and the rate constant was extracted from the fitting results.

The activation energy (*E_a_*) of each sample was determined by first extracting rate constants at different temperatures for each sample and then treating the data obtained with the Arrhenius equation (Equation (3)) below:

(3) $$\ln k=-\frac{E_{a}}{RT}+A$$

where *T* is temperature, *R* is a gas constant, *A* is a constant, and *k* is a rate constant. Plotting ln*k* with the reciprocal of temperature 1/*T* and fitting the results gives *E_a_*.

## 3. Results and Discussion

### 3.1. Polysiloxane Synthesis and Characterization

**Scheme 1:**
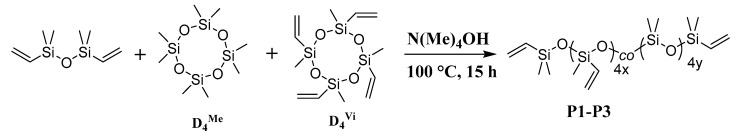
Preparation of vinyl silicone oils via ring-opening polymerization.

Vinyl polysiloxanes (P1-P3) with different vinyl group contents were prepared via the copolymerization of D_4_^Me^ and D_4_^vi^ by anionic ring-opening polymerization catalyzed by N(Me)_4_ OH (Scheme 1).

**Figure 1:**
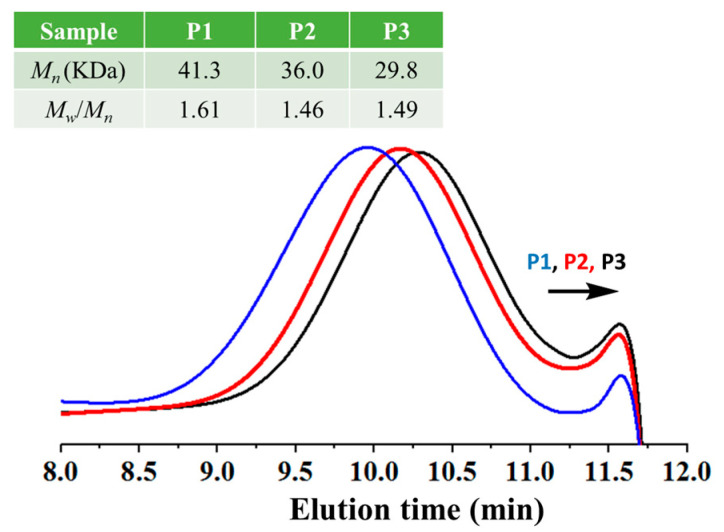
Gel permeation chromatography (GPC) traces of methyl vinyl silicone oils P1-P3.

The obtained products were narrowly distributed (Figure 1) with a polydispersity index (PDI) around 1.5 and featured compositions in good agreement with the theoretical values as revealed by the NMR analysis (Figures S3–S5). The mole ratio of D_4_^Me^ to D_4_^vi^ was quantified by ^1^H-NMR integration with P2 and P3. Methyl signals from the dimethyl units (0.07 ppm) and methyl vinyl (0.0 ppm) units were separately integrated and compared to calculate the ratio for each unit.

### 3.2. Uptake Performance in Pure H_2_ Atmosphere

**Figure 2:**
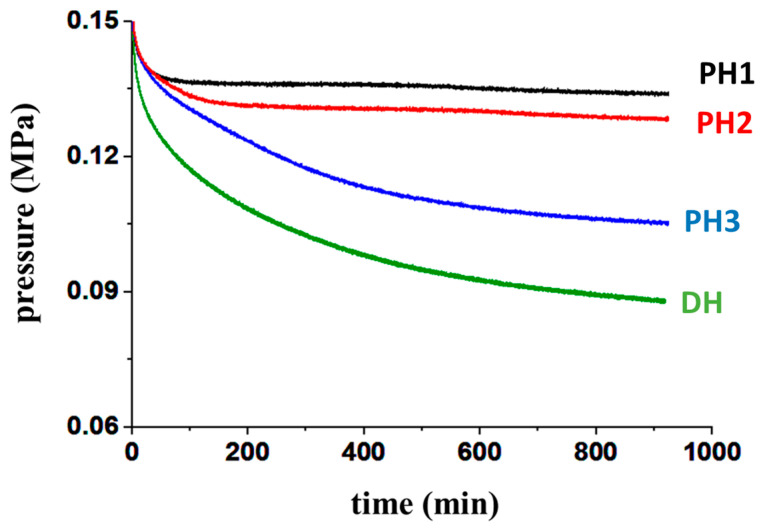
H_2_ pressure vs. time for PH*x* at 298 K and an initial pressure of 200 KPa.

To test the capacity (volume of hydrogen absorbed per gram of getter, mL/g) and sensitivity of the vinyl silicone oils obtained, absorption tests under both pure and diluted hydrogen atmosphere conditions were carried out. Test samples (PH1-PH3) were prepared by mixing silicone oil P1-P3 with Pd/C at a weight ratio of 3:1. For testing in pure hydrogen, the paste-like mixture was loaded into a stainless-steel container, where the container was evacuated and the sample was then brought into contact with hydrogen, where the pressure drop in the container was monitored over time. Figure 2 shows pressure decay curves against time for all three samples. Note that the initial pressure drop observed at the beginning of the experiment was due to an expansion of gas from the gas reservoir to the reactor.

The results with the getter sample (DH) prepared by mixing D_4_^vi^ with Pd/C at a weight ratio of 3:1 are also shown for comparison.

The uptake capacity of each polymer getter was calculated from the pressure drop (Figure 2) and the results are shown in Table 1.

Since the vinyl content of each sample could be accurately determined by titration, the volume of hydrogen scavenged by each sample could also be calculated based on the consumption of vinyl groups during hydrogenation. As seen from the results, the two calculated capacities are in good agreement with each other, suggesting the H_2_ absorption with a Pd/C catalyst was negligible in the test condition. The DH getter had the highest capacity, and the capacities of the polymer getters (PH1-PH3) were lower than the DH getter and decreased with a decrease in vinyl content. Among the samples tested, DH and PH3 were of almost the same chemical composition, and the lower capacity of PH3 can be attributed to the reduced mobility of polymer chains. The addition of H_2_ to unsaturated double or triple bonds is governed by efficient contact of the reaction site with the catalyst [17], where hydrogen atoms impinged on Pd clusters diffuse in an order of several micrometers and recombine if no reaction sites are available [18,19]. Polymers with long chains have restricted mobility and thus are less likely to have complete contact with a palladium cluster, which ultimately leads to a lower degree of hydrogenation. Figure 2 also shows that D_4_^vi^ reacts far faster (as revealed by the initial slope of each curve) than PH3, suggesting that the reaction rate is likely dominated by the diffusion and migration of H_2_ into the sample at the early stage of the reaction. It is noteworthy that although the DH getter had the highest capacity, it is volatile as D_4_^vi^ has boiling point of about 224 °C. The volatilization of D_4_^vi^ not only causes getter loss, but it also contaminates the application environment, so it has no practical value when compared with polymer getter PH3. Since the PH getters are not cross-linked, the palladium catalyst can be recycled the end of use by washing polysiloxane with an organic solvent.

### 3.3. Absorption under Low Hydrogen Partial Pressure

Since the explosion limit of hydrogen in air is 4%, a useful getter needs to be reactive to a low concentration of H_2_. To test the sensitivity values of the getters, we performed absorption tests in 1% and 5% H_2_ in N_2_. The concentration change of H_2_ was measured over time by gas chromatography and the results are shown in Table 2. All the data shown have been normalized with the H_2_ concentration in the reaction chamber without a sample.

Inspection of the results shows that the H_2_ concentration decreased with time under both test conditions, suggesting the gradual addition of H_2_ to the getters. Samples with a higher vinyl content had a higher reaction rate and the reaction accelerated as the initial H_2_ concentration increased from 1% to 5%. This came as no surprise, as chemical reaction is usually dominated by collision, where reaction in a more concentrated condition accelerates as the odds of collision improve. H_2_ could be effectively eliminated to very low levels in all experiments, suggesting that these newly developed polymers are ideal getter candidates.

### 3.4. Synthesis and Characterization of Improved Getters

**Scheme 2:**
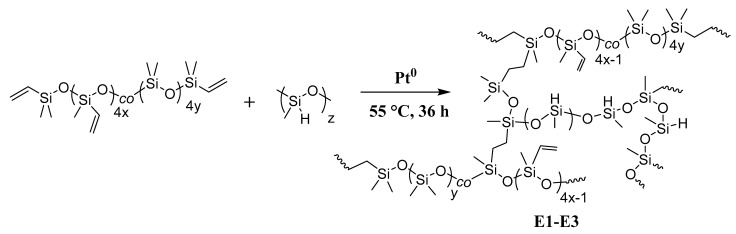
Cross-linking of PH*x* by hydrosilication catalyzed by Pt^0^.

**Figure 3:**
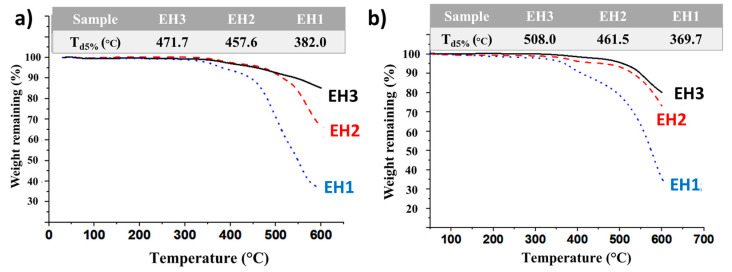
TGA curves of getter elastomer EH*x* (*x* = 1, 2, 3) in N_2_ (**a**) and in air (**b**).

We prepared a cured getter by cross-linking PH*x* (*x* = 1, 2, 3, Scheme 2) with polymethylhydrosiloxane. Thermoset getters feature shape stability and are adaptable to various scenarios where getter flexibility is required. By adding 2 wt % poly methylhydrosiloxane and a catalytic amount of Pt^0^ catalyst into PH*_x_*, soft black elastomers were obtained after curing at 65 °C for 3 days. Thermogravimetric Analysis (TGA) measurements of these getters showed that they were quite thermostable with 5 wt % weight loss (T_d5%_) higher than 382.0 °C in nitrogen and 369.7 °C in air (Figure 3). In both air and nitrogen atmospheres, weight loss decreased as vinyl content increased, where samples with a higher vinyl group content had both higher T_d5%_ and char yield. This trend has been observed before and is attributed to the stabilizing effects of vinyl groups [20,21].

**Figure 4:**
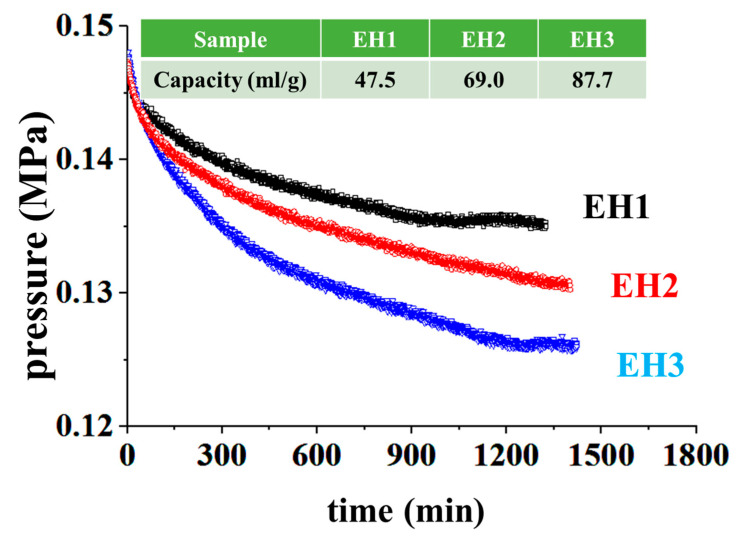
H_2_ pressure vs. time plot for EH*x* at 298 K and an initial pressure of 200 KPa. Inset shows the capacity of samples EH1-EH3.

Stress-strain testing of the samples demonstrated a maximum tensile strength of about 0.4 MPa and a break elongation of about 120% (Table S1). Reactivity of the cured getters was also evaluated by testing in pure and dilute hydrogen atmospheres. Compared with the results for the PH*x* samples, the capacities of all EH*x* samples were reduced (Figure 4).

Since the consumption of vinyl groups from cross-linking was less than 3 wt %, even when 100% conversion was assumed for the hydrosilication reaction, the reduction in capacity was mainly caused by the restricted contact of reaction sites with a Pd catalyst. As the vinyl content increases from PH1 to PH3, the odds of reaction site and catalyst contact rapidly decrease, and this explains why the capacity of EH1 is close to PH1, while the capacity difference between PH3 and EH3 is huge. As opposed to the testing results in pure H_2_, the results in a dilute H_2_ atmosphere were not too different from the original and cured getters (Table S2). Under a dilute H_2_ atmosphere, getters were present in an excessive amount (16 eqv. for EH1), vinyl consumption was less than 7%, and thus similar uptake results were observed.

### 3.5. Kinetic Study

**Figure 5:**
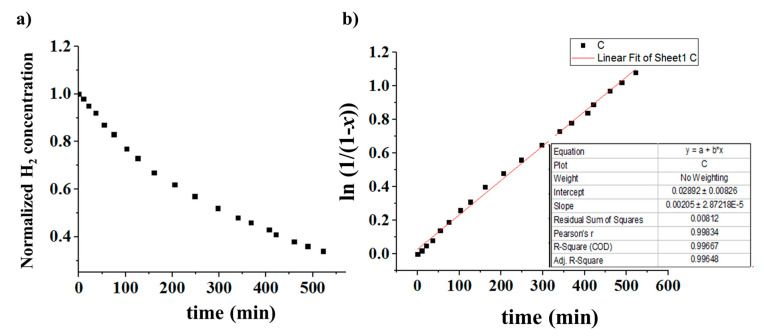
(**a**) Plot of normalized H_2_ concentration against time at 273 K. (**b**) Plot of ln(1/(1-*x*)) with time and linear data fitting.

Kinetic studies were performed to explore the temperature dependence of H_2_ absorption. Absorption tests at different temperatures (0–60 °C) were performed for a series of three samples (DH, PH2, and EH2) in 1% H_2_ in N_2_. The activation energy (*E_a_*) of hydrogenation for each sample was obtained by the method described in Section 2.8. The data for PH_2_ were used to illustrate this process. Normalized concentrations of H_2_ at different times at 273 K were recorded during hydrogenation via GC (Table S3). The obtained data were then used to construct the time–concentration curve shown in Figure 5a. Plotting the natural logarithm of the reciprocal of the normalized H_2_ concentration against time and fitting the results provides a reaction rate constant (Figure 5b) of 273 K.

All the absorptions showed first-order characteristic when the H_2_ consumption was lower than about 30%. Rate constants were extracted from the initial slopes (H_2_ conversion <30%) of these curves. Rate constants (Tables S4 and S5) at each test temperature changed in the order of DH > PH2 > EH2. This tendency may either result from restricted contact with a catalyst as the mobility of the chain is progressively reduced from the monomer to the network or slower diffusion of H_2_ in more viscous media. The plot of the rate constants at different temperatures with the reciprocal of temperature shows an Arrhenius correlation (Figures S6–S8). The plotting of ln*k* as a function of 1000/*T* is shown in Figures S6–S8, where the activation energy was calculated by multiplying the slope of the fitting equation with the gas constant (R = 8.31 J∙K∙mol^–1^). The activation energies extracted from the fitting results are shown in Table 3. The results are almost identical, suggesting that the mechanism of H_2_ addition to vinyl groups is not altered by polymerization and curing.

### 3.6. Compounded Getter Synthesis and Evaluation

The capacities of the cured getters could be further improved by the addition of DEB. By adding different amounts (50 wt %, 60 wt %, and 70 wt %) of a DEB getter into P3 and curing the mixture, this provided compounded getters (CG1-CG3) with increased capacity (Table 4).

CG getters have capacities comparable to commercially available DEB getters with a capacity ranging from 145 mL/g to 223 mL/g. CG getters are also flexible and are compatible with environments featuring complex configurations. The hydrogen scavenged by the vinyl polymer in CG getters was calculated by the deduction of the DEB getter contribution from the total capacity. The polymer contribution counter-intuitively increases as its ratio decreases from 50 wt % to 30 wt %. Catalyst loading increases with greater DEB getter proportions (Table 4), which leads to more effective contacts between vinyl groups and catalyst sites and the observed increased conversion of vinyl groups.

## 4. Conclusions

Novel polysiloxane hydrogen getters were prepared and studied in this work. The capacities of these new materials are dominated by their structures and compositions, where uncured paste getters have higher capacity compared with cured getters as crosslinking rapidly reduces the possible contact of the vinyl group with the catalyst; however, the disadvantage of the cured getter is supplemented by its flexibility and compatibility with environments featuring a complex structure. A compounded getter with both shape stability and an optimal capacity can be obtained by adding an organic DEB getter into vinyl polysiloxane and then curing the mixture. Although the costs of these getters are higher due to the addition of DEB, the getters are useful in scenarios where shape stability, and high capacity are required. Due to the outstanding performance of the compounded getter, future work should be conducted in an effort both to build new getter elastomers with high capacity and resolve the structure–property relationship for the new material.

## Figures and Tables

**Table 1:** (a) Getter capacity determined by both a pressure drop test and (b) chemical titration. (c) Vinyl content of each sample before and (d) after H_2_ absorption.

Sample	Capacity (mL/g)		Vinyl Content (mmol/g)	
PH1	52.6 ^a^	53.9 ^b^	2.7 ± 0.15 ^c^	0.3 ± 0.11 ^d^
PH2	104.6	107.8	5.8 ± 0.25	1.0 ± 0.14
PH3	202.5	211.2	10.8 + 0.32	1.4 ± 0.18
DH	237.2	247.1	11.5 ± 0.43	0.5 ± 0.12

**Table 2:** Normalized H_2_ concentration with time for PH1-PH3 under 1% and 5% H_2_ test conditions.

	1% H_2_ in N_2_				5% H_2_ in N_2_			
Sample	11 min	40 min	120 min	240 min	8 min	25 min	70 min	120 min
PH1	85.8%	58.7%	12.2%	0.8%	74.7%	29.8%	6.3%	<10 ppm
PH2	64.2%	40.8%	3.1%	<10 ppm	50.7%	23.0%	1.2%	<10 ppm
PH3	48.6%	24.8%	0.1%	<10 ppm	18.6%	7.8%	<10 ppm	<10 ppm

**Table 3:** The activation energy for the hydrogenation of DH, PH2, and EH2.

Sample	DH	PH2	EH2
*E_a_* (KJ/mol)	20.0	20.1	19.6

**Table 4:** (a) Capacities of compounded getters. (b) Pd loading of a compounded getter. (c) Polymer contribution to a compounded getter with a weight of 1.0 g. (d) Vinyl group conversion in each compounded getter.

Sample	CG1	CG2	CG3
Total capacity (mL/g) ^a^	133	164	210
Catalyst loading (wt %) ^b^	5.3	6.3	7.4
Polymer contribution (mL) ^c^	25.0	34.4	58.8
Vinyl conversion ^d^	19.2%	32.6%	75.4%

## Data Availability

Data is contained within the article.

## Supplementary Materials

The following are available online at https://www.mdpi.com/article/10.3390/ma14081853/s1, Figure S1: Schematic illustration of pure hydrogen test apparatus, Figure S2: Schematic illustration of mixture gas test apparatus, Figure S3: ^1^H NMR spectrum of P3 in CDCl_3_, Figure S4: ^1^H NMR spectrum of P2 in CDCl_3_. Mole ratio of vinyl unit calculated from the integration is 47.9%, which is in good agreement with the theoretical value 46.6%, Figure S5: ^1^H NMR spectrum of P1 in CDCl_3_. Mole ratio of vinyl unit calculated from the integration is 24.4%, which is in good agreement with the theoretical value 22.0%, Figure S6: Arrhenius plot of PH2 with linear fit, Figure S7: Arrhenius plot of EH2 with linear fit, Figure S8: Arrhenius plot of DH with linear fit, Table S1: Maximum stress (a) and elongation at break (b) of EH*x* getter, Table S2: Normalized H_2_ concentrations with time for EH*x* in H_2_/N_2_ mixture, Table S3: Normalized H_2_ concentrations at different time intervals at 273 K for PH2, Table S4: Rate constant of PH2 at different temperature, Table S5: Rate constant of DH (a) and EH2 (b) at different temperature.
