# Supplementary material for: Preparation and Characterization of a Novel Vinyl Polysiloxane Getter for Hydrogen Elimination
Source: Materials (Basel). 2021 Apr 8;14(8):1853. doi: 10.3390/ma14081853 (PMC8068371; doi:10.3390/ma14081853)
Supplement: Supplementary file 1 [file materials-14-01853-s001.zip › materials-1161182-supplementary.pdf]

materials

Article

Preparation and Characterization of a Novel Vinyl Polysiloxane Getter for Hydrogen Elimination

Tao Xing [1,*,†], Yong Xu [1,†], Juying Wu [1], Yu Wang [2] and Lifeng Yan [2,*]

[1] Institute of System and Engineering, China Academy of Engineering Physics, 64 Mianshan Road, Mianyang 621900, China; xuy@caep.cn (Y.X.); wujy@caep.cn (J.W.)

[2] Department of Chemical Physics, University of Science and Technology of China, Hefei 230026, China; wy1998@mai.ustc.edu.cn

* Correspondence: 412xingt@caep.cn (T.X.); lfyan@ustc.edu.cn (L.Y.)

† These authors contribute equally to this article.

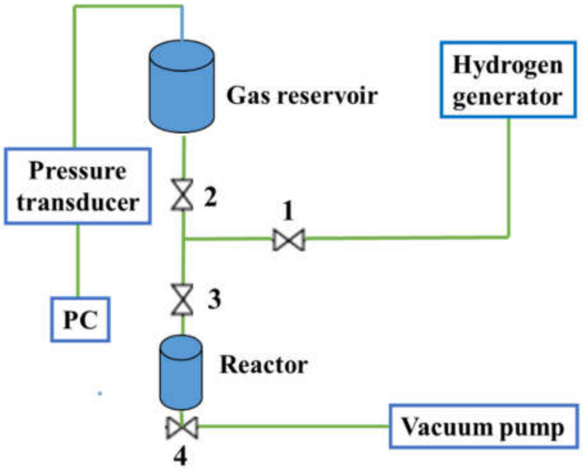

Figure S1. Schematic illustration of pure hydrogen test apparatus.

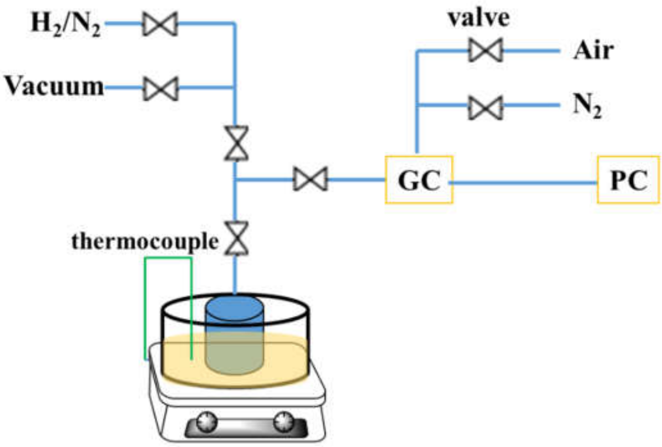

Figure S2. Schematic illustration of mixture gas test apparatus.

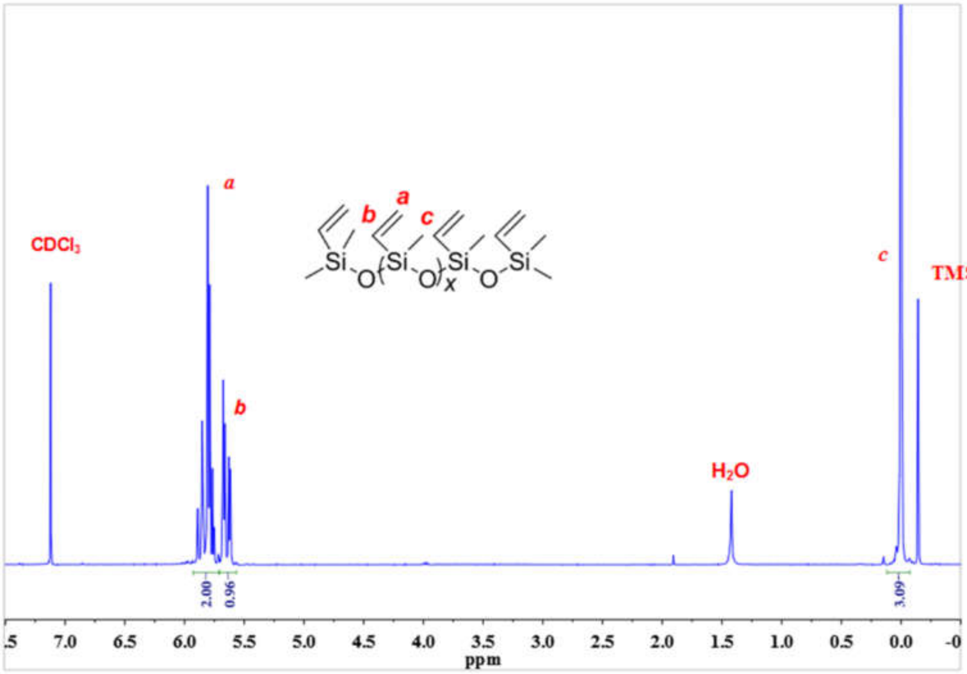

Figure S3. ^{1}H NMR spectrum of P3 in CDCl3.

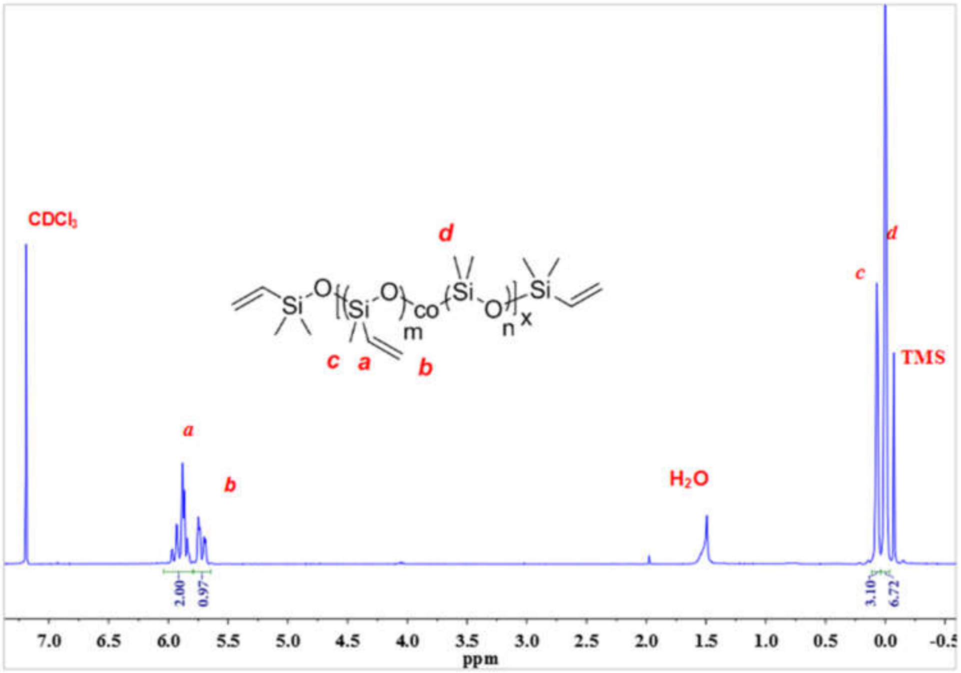

Figure S4. ^{1}H NMR spectrum of P2 in CDCl3. Mole ratio of vinyl unit calculated from the integration is 47.9%, which is in good agreement with the theoretical value 46.6%.

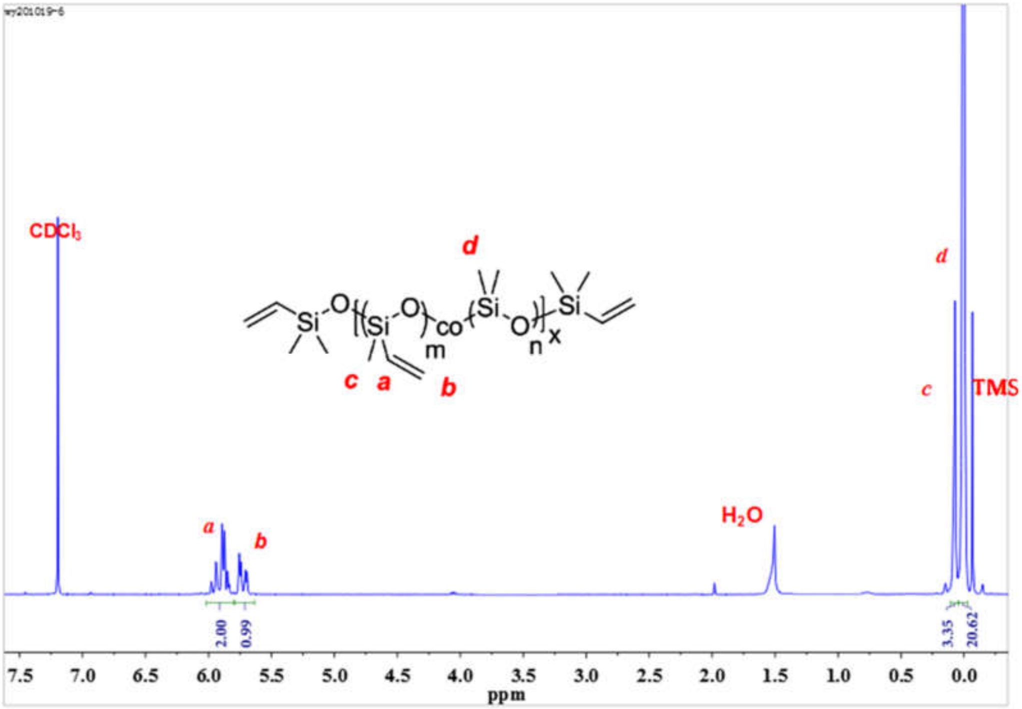

Figure S5. ^{1}H NMR spectrum of P1 in $CDCl_3$. Mole ratio of vinyl unit calculated from the integration is 24.4%, which is in good agreement with the theoretical value 22.0%.

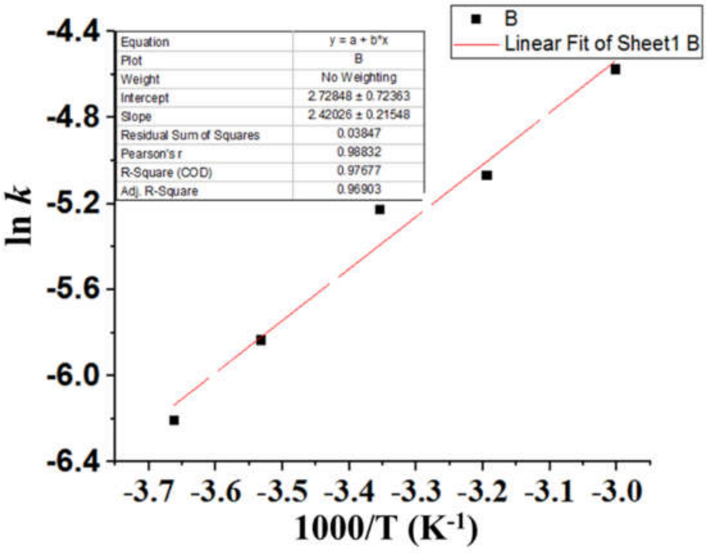

Figure S6. Arrhenius plot of PH2 with linear fit.

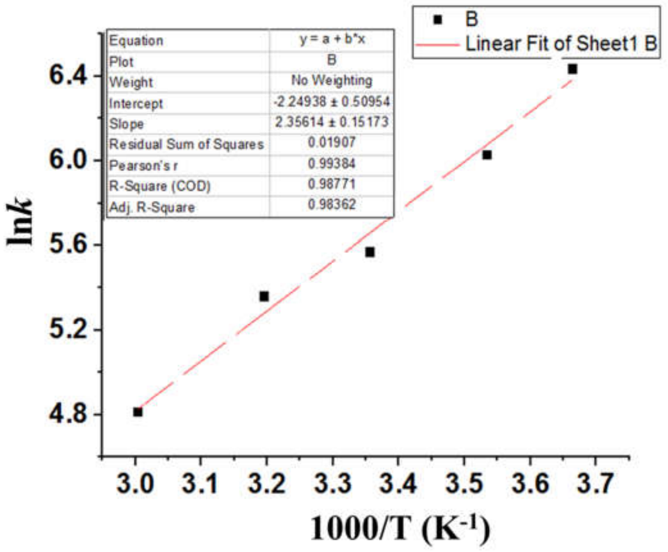

Figure S7. Arrhenius plot of EH2 with linear fit.

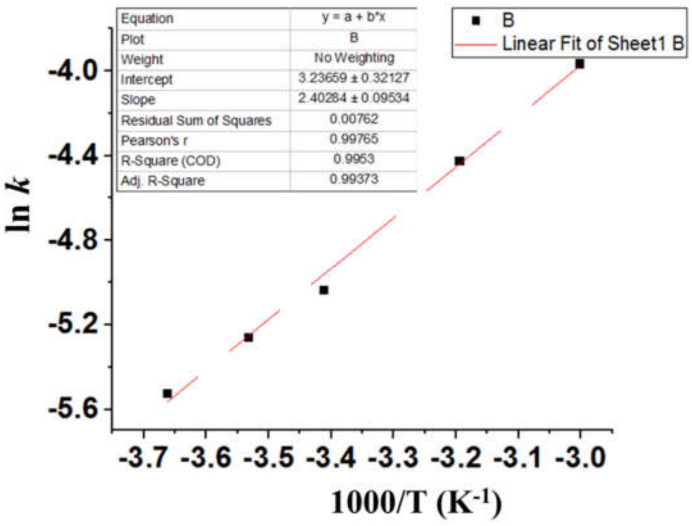

Figure S8. Arrhenius plot of DH with linear fit.

Table S1. Maximum stress (a) and elongation at break (b) of EHx getter.

Getter	EH1	EH2	EH3

Copyright: © 2021 by the authors. Licensee MDPI, Basel, Switzerland. This article is an open access article distributed under the terms and conditions of the Creative Commons Attribution (CC BY) license (http://creativecommons.org/licenses/by/4.0/).

σ^a (MPa)	0.41	0.37	0.54
$\boldsymbol{\varepsilon}^{\mathbf{b}}$	117%	125%	118%

Table S2. Normalized H_2 concentrations with time for EH*x* in H_2/N_2 mixture.

	1% H_2 in N_2				5% H_2 in N_2			
Sample	1 h	5 h	7 h	12 h	1 h	5 h	8 h	12 h
EH1	78.3%	21.5%	17.8%	1.3%	74.4%	15.0%	11.5%	< 10 ppm
EH2	76.3%	19.0%	8.3%	< 10 ppm	65.0%	13.5%	8.1%	< 10 ppm
EH3	64.5%	16.7%	8.0%	< 10 ppm	57.2%	9.5%	4.3%	< 10 ppm

Table S3. Normalized H_2 concentrations at different time intervals at 273 K for PH2.

Time (min)	1-x	$\ln\frac{1}{1-x}$
0	1	0.00
10	0.98	0.02
21	0.95	0.05
36	0.92	0.08
54	0.87	0.14
75	0.83	0.19
102	0.77	0.26
126	0.73	0.31
161	0.67	0.40
205	0.62	0.48
248	0.57	0.56
297	0.52	0.65
340	0.48	0.73
368	0.46	0.78
407	0.43	0.84
421	0.41	0.89
460	0.38	0.97
489	0.36	1.02
522	0.34	1.08

Table S4. Rate constant of PH2 at different temperature.

T (K)	273	283	298	313	333
k (s^{-1})	0.0021	0.0029	0.0054	0.0063	0.0103

Table S5. Rate constant of DH (a) and EH2 (b) at different temperature.

T (K)	273	283	298	313	333
k (s^{-1})^a	0.0041	0.0052	0.0065	0.012	0.0193
k (s^{-1})^b	0.0016	0.0024	0.0038	0.0047	0.0081
